# Recent Advances and Challenges in Uveal Melanoma Immunotherapy

**DOI:** 10.3390/cancers14133094

**Published:** 2022-06-23

**Authors:** Yihang Fu, Wei Xiao, Yuxiang Mao

**Affiliations:** 1 State Key Laboratory of Ophthalmology, Zhongshan Ophthalmic Center, Sun Yat-sen University, Guangzhou 510060, China; fuyh8@mail2.sysu.edu.cn; 2 Guangdong Provincial Key Laboratory of Ophthalmology and Visual Science, Guangzhou 510060, China; 3 Guangdong Provincial Clinical Research Center for Ocular Diseases, Guangzhou 510060, China

**Keywords:** immune checkpoint inhibitors, HDAC inhibitors, oncolytic virus, tebentafusp, uveal melanoma, immunotherapy

## Abstract

**Simple Summary:**

Uveal melanoma is the most common primary intraocular malignancy in adults. Although it can be controlled locally, half of the patients still develop metastases. To date, there have been no standard therapeutic strategies for the prevention or treatment of metastases. Existing therapies, such as chemotherapy and targeted therapies, induce only minimal responses. This review focuses on newly published research on immunotherapy. We highlight expanding treatments and their clinical outcomes, as well as propose promising new treatments and feasible checkpoints. Based on these findings, we provide innovative insights into feasible strategies for the treatment of patients with uveal melanoma.

**Abstract:**

Uveal melanoma (UM) is the most common primary intraocular malignancy in adults. Compared to cutaneous melanoma (CM), which mainly harbors *BRAF* or *NRAS* mutations, UM predominantly harbors *GNAQ* or *GNA11* mutations. Although primary UM can be controlled locally, approximately 50% of patients still develop metastases. To date, there have been no standard therapeutic strategies for the prevention or treatment of metastases. Unfortunately, chemotherapy and targeted therapies only induce minimal responses in patients with metastatic UM, with a median survival time of only 4–5 months after metastasis detection. Immunotherapy agents, such as immune checkpoint inhibitors, have achieved pioneering outcomes in CM but have shown limited effects in UM. Researchers have explored several feasible checkpoints to identify options for future therapies. Cancer vaccines have shown little in the way of therapeutic benefit in patients with UM, and there are few ongoing trials providing favorable evidence, but adoptive cell transfer-related therapies seem promising and deserve further investigation. More recently, the immune-mobilizing monoclonal T-cell receptor against the cancer molecule tebentafusp showed impressive antitumor effects. Meanwhile, oncolytic viruses and small molecule inhibitors have also gained ground. This review highlights recent progress in burgeoning treatments and provides innovative insights on feasible strategies for the treatment of UM.

## 1. Introduction

Uveal melanoma (UM) is the most common primary intraocular malignancy in adults, despite its low incidence [1]. It originates from ocular melanocytes of the choroid (approximately 85%), ciliary body (5–8%), or iris (3–5%) and mainly occurs unilaterally in the posterior pole [1,2]. There is overlap in the cell-of-origin and some risk factors between UM and cutaneous melanoma (CM), however, the major environmental risk factor for CM, UV-light exposure, cannot induce UM [3]. It is worth highlighting that UM has no known environmental risk factors. UM generally harbors *GNAQ* or *GNA11* mutations distinct from the *BRAF* or *NRAS* mutations in CM [2,4]. The incidence ranges from 1 to 9 per million people per year and no obvious sex susceptibility has been documented [5]. Most patients with UM are diagnosed between the 5th and 7th decades of life with a median age of 62 years [6,7]. The 5-year and 15-year disease-related mortalities are ~30% and 45%, respectively [8]. Factors including light eye color, fair skin, melanocytoma, congenital ocular melanocytosis, neurofibromatosis, dysplastic nevi, and the BAP1-tumor predisposition syndrome predispose individuals to UM [9,10,11].

The long-term prognosis of UM is poor, and approximately half of the patients still suffer from metastases, irrespective of treatment for the primary tumor [8,12]. Metastatic UM, which has a poor response to chemotherapy or targeted therapies, is usually fatal a year after diagnosis. The 1-year and 2-year overall survival (OS) rates are 43% and 8% after metastasis detection, respectively [13,14]. The liver is the most common metastatic site, but other metastatic sites include the lungs, brain, skin, bone, and lymph nodes, and the main route of spread is hematological. Several clinical and histological factors are associated with metastasis, including cell type, pigmentation, tumor size, ciliary body involvement, and intra- and extra-scleral extension [15].

Genetic variations also play important roles; extra gains of chromosome 8q and/or heterozygotic losses of chromosome 3 typically predict poorer prognosis, whereas extra gains of chromosome 6p imply better survival [16,17]. There are several significant mutations, including *GNAQ*, *GNA11*, *SF3B1*, *EIF1AX*, *BAP1*, *CYSLTR2*, *SRFF2*, *MAPKAPK5*, and *PLCB4* [18]. Though the detailed molecular pathways of developing metastatic UM have not been discovered yet, *SF3B1* and *BAP1* mutations correlate with late and early metastasis, respectively, whereas *EIF1AX* mutations have been associated with low metastatic risk and favorable survival [19,20]. Among these mutations, *BAP1* mutations are the most notable. The *BAP1* gene, which encodes BRCA1-associated protein 1 (BAP1), is located on chromosome 3 and has tumor suppressor activity [21]. A study showed that 87% of monosomy 3 UM patients have *BAP1* alterations, while others have decreased mRNA expression of *BAP1* [17]. Furthermore, monosomy 3 UM with *BAP1* loss also exhibits a different pattern of methylation, which might lead to extensive epigenetic modifications [17]. Therefore, based on various UM tumor parameters such as the number of chromosome copies, expression of mRNA, microRNA, and long non-coding RNA, as well as patterns of methylation, the Cancer Genome Atlas divided UM into four subgroups, named A, B, C, and D by Jager et al. [17]. Specifically, subgroup A is characterized by normal 8q, disomy 3, and additional 6p with a favorable prognosis; B is characterized by partial extra 8q, disomy 3, and additional 6p with late metastases; and C and D both have monosomy 3 and an unfavorable prognosis, in which C gains only one extra 8q, but D gains more than one extra 8q [17]. Robertson et al. [18] identified four subtypes: two disomy 3, which carry *EIF1AX* or *SF3B1* alterations, were connected to low metastatic risk and better prognosis, two monosomy 3 subtypes, which carry *BAP1* alterations, were connected to high metastatic risk and poor prognosis. Harbour et al. [22,23] divided primary UM into two classes by a gene expression profile containing 12 discriminating genes: class 1 with low metastatic risk and class 2 with high metastatic risk.

Furthermore, epigenetic alterations, such as non-coding RNA aberrations, histone modifications, and DNA methylation, play essential roles in the development of UM. The non-coding RNAs are crucial biological regulators and participate in nearly all biological processes [24]. Several microRNAs (miRNAs) demonstrate different levels of expression in UM. For instance, miR-20a, which promotes the proliferation of tumor cells, is highly expressed in patients with UM, while the tumor suppressor miR-34 family shows significantly decreased expression [25,26]. It was also found that 329 long non-coding RNAs are expressed differentially in UM [27]. As for histone modifications, both histone acetylation and methylation have a great impact on the development of UM. H3K14ac, an important pattern of histone acetylation, promotes the development of UM through its downregulation [28]. The histone deacetylases (HDACs), which exist in UM tissues extensively, can inhibit the expression of cancer suppressor genes [29]. The histone methylation can regulate immune response and promote gene expression in UM. In regard to DNA methylation, hypomethylation and hypermethylation would affect the development of UM in multiple ways, such as by activating the cell cycle, repairing impaired DNA, and enhancing the RAS signal [24]. Moreover, RNA modifications and chromosome conformations also have impact on the tumorigenesis of UM.

The main purpose of treating primary UM is to prevent distant metastasis and preserve the globe and vision. Despite the absence of a standardized management algorithm or care pathway, several therapies can control the growth of the local tumor and even conserve the vision of the affected eye [30,31]. These include radiotherapies such as brachytherapy (plaque radiotherapy with Ru106 or I125), proton beam radiotherapy and stereotactic radiotherapy, and local resection [1,32]. Enucleation and orbital exenteration are necessary in patients with massive tumors [1,32].

Currently, no preventive or curative treatment is available for metastatic UM. Close systemic surveillance such as liver imaging (hepatic MRI) is recommended. Patients with metastatic diseases were encouraged to participate in clinical trials [32,33]. Despite limited outcomes, some therapeutic approaches are available, such as surgery, hepatic intra-arterial chemotherapy, transarterial chemoembolization, isolated or percutaneous hepatic perfusion, and selective internal radiation therapy [34].

Burgeoning therapies for advanced CM, especially immune checkpoint inhibitors (ICIs), have achieved impressive progress in the past decades. Nevertheless, such treatments have shown minimal benefits in terms of improving the prognosis of UM. Distinctions in tumor biology, genetics, tumor microenvironment, and the comparative clinical behavior of UM and CM shows that the development of therapeutic strategies can refer to successful instances of CM treatment but should also take into consideration the specific characteristics of UM [35,36].

## 2. Immune Escape and Immunosuppressive Micro-Environment of UM

As an immunologically privileged organ, the eye offers a growth advantage to primary UM via multiple physiological mechanisms, such as the blood−eye barrier, various immunosuppressive molecules, and constructive expression of the FAS ligand [37,38]. Uveal melanoma can mimic mechanisms that contribute to immune privilege to protect itself in both the eye and metastatic sites and, consequently, achieve immune escape and systematic dissemination [39].

Uveal melanoma can inhibit both innate and adaptive immunity. Innate immunity primarily involves two cell types: natural killer (NK) cells and macrophages. NK cells can eliminate tumor or virus-infected cells [40]. In UM, human leukocyte antigen-E (HLA-E), which combines with the inhibitory receptor of NK cells, CD94/NKG2, was found to be overexpressed, resulting in the silencing of the cytolysis procedure mediated by NK cells [39,41]. In addition, the local secretion of tumor growth factor β inhibits the function of NK cells and alters their susceptibility to UM cells [42,43]. Macrophages are divided into the M1 subtype, which acts as an antigen-presenting cell (APC) and plays an active part in the immunostimulatory process, and the M2 subtype, which inhibits inflammatory and immune responses by downregulating pro-inflammatory cytokines and participates in the immunological tolerance process [44]. In UM, tumor-associated macrophages (TAMs) are predominantly M2 and thus, the immune process is greatly suppressed [44,45,46].

Adaptive immunity is composed of humoral immunity and cell-mediated immunity. In UM, T-lymphocyte-mediated cellular immunity mainly plays a role. CD8+ cytotoxic T-cells (CTL) or CD4+ helper T-cells are inhibited by UM cells in multiple ways [38,47]. For instance, indoleamine 2, 3-dioxygenase (IDO) can catalyze tryptophan degradation, which is crucial for the activation and proliferation of T-cells. Hence, this might result in T-cell impairment and immune escape [48]. The upregulation of programmed death ligand-1 (PD-L1) induced by interferon-gamma (IFN-γ) can inhibit T-cell activation [49]. IFN-γ can suppress the destructive function of CTLs and contribute to UM cell resistance to perforin-mediated cytolysis [39,50].

In summary, immunotherapy with UM should target immune characteristics. Due to immune inhibition, active development of cancer vaccines and enhancement of T-cell response or function, such as adoptive cellular therapy or novel monoclonal molecule reagents, are critical to strengthen the anti-tumor effect. In addition, based on the particular molecular mechanisms of UM, specific UM-related receptors or proteins need to be given attention to optimize existing treatment strategies and seek more promising targeted checkpoints. Herein, we review recent literature on UM immunotherapy and propose therapeutic approaches.

## 3. ICIs

### 3.1. Cytotoxic T-Lymphocyte-Associated Antigen-4 Inhibitors: Ipilimumab and Tremelimumab

As the first drug approved for metastatic CM therapy by the Food and Drug Administration (FDA) in 2011, ipilimumab overcomes the immunoinhibitory effect by blocking the immune checkpoint molecule cytotoxic T-lymphocyte-associated antigen-4 (CTLA-4), which downregulates T-cell activation [2,51,52].

Previous studies have demonstrated that ipilimumab leads to limited degrees of stable disease (SD), partial response (PR), or rare complete response (CR) in metastatic UM patients in studies with small sample sizes. The first retrospective study involving 20 patients with metastatic UM showed a 5-month median OS after ipilimumab intervention [53]. Another retrospective population-based study compared the efficacy of monotherapy with a combination of ICIs [54]. The median OS was 9.9 months, which is not superior to recent estimates of median OS of UM (10.2 months) [14]. None of the 24 patients treated with ipilimumab responded to the intervention or achieved PR or CR [54]. A phase Ib/II trial on combined radiofrequency ablation and ipilimumab treatment also suggested that ipilimumab was less promising and the trial did not induce effective responses despite good tolerance [55]. Similar results were seen in a retrospective multicenter study, in which none of the 11 patients with UM achieved CR or PR and only two achieved SD (18.2%) [56].

Tremelimumab (CP 675206) is a human monoclonal antibody that targets CTLA-4. A phase II study of 11 patients with UM receiving tremelimumab showed a 2.9-month median progression-free survival (PFS) and a 12.8-month median OS [57]. The median OS reported in a study of over 700 patients with UM ranged from 3 to 4 months after diagnosis [58]. None of the patients achieved CR or PR, resulting in the cessation of the study at the first interim stage (Table 1) [57].

Overall, the clinical effects of CTLA-4 inhibitors are unfavorable for metastatic UM. However, they may act as feasible treatments for a small portion of patients with UM. To date, factors influencing individual responses to ipilimumab are unknown [59].

### 3.2. PD 1/PD-L1 Inhibitors: Pembrolizumab, Nivolumab, and Atezolizumab

These inhibitors function by blocking the specific interaction of the PD-1/PD-L1 ligand receptor, thereby overcoming the immune inhibition of T-cells [60]. Pembrolizumab, nivolumab, and atezolizumab (monoclonal antibodies targeting the PD-1/PD-L1 pathway at different sites) have been approved for treating melanoma. The investigation of their therapeutic efficacy was mainly based on several small, retrospective studies rather than controlled prospective trials, so the outcomes should be read with caution.

A previous study illustrated that nivolumab contributed to improved OS and PFS in patients with metastatic melanoma by recovering cytokine production and T-cell activation [61]. Recently, a single institution study involving 14 patients with metastatic UM reported an overall response rate (ORR) of 7.1% [62]. The median PFS and OS were 10 and 60 weeks, respectively, and the tolerability of nivolumab was generally good [62]. Another multicenter study showed an ORR of 18% in 17 patients with metastatic UM, together with 5.8 months for median PFS and 10.5 months for OS [63]. Grade 1 and 2 adverse events (AEs), such as decreased appetite and fatigue, were the most common treatment-related side effects (17%); no grade 3 or 4 AEs were observed [63].

Concerning pembrolizumab, two former expanded access programs have shown certain gaps in ORR and PFS, along with an unknown median OS [64,65]. In a recent small single-arm phase II study (NCT02359851) (Table 2), the 11-month median PFS was much longer than the former, together with an unknown median OS [66]. Another prospective cohort single arm study showed two patients with PR (11.7%) and six with SD (35.3%) among 17 patients with metastatic UM, with a 3.8-month median PFS and an unknown median OS [67]. Two studies conducted by Bol et al. [54] and Jansen et al. [68], which enrolled 43 and nine patients with UM, respectively, had similar clinical results. None of the patients achieved CR. Bol et al. reported three patients with PR (7%) and 12 with SD (27.9%), and Jansen et al. reported five patients with SD (56%) [54,68]. The median PFS were 4.8 months (approximately 144 days) and 18 weeks (approximately 126 days), respectively, which were comparable to the results of Rossi et al. [67], and the median OS were 10.3 months (approximately 309 days) and 46 weeks (approximately 322 days), respectively [54,68].

Previous studies have compared the clinical outcomes of different anti-PD-1 and anti-PD-L1 antibodies. Algazi et al. [69] conducted an analysis of 56 patients with metastatic UM, in which 38 were treated with pembrolizumab, 16 with nivolumab, and 2 with atezolizumab. The ORR was 3.6%, the median PFS was 2.6 months, and the median OS was 7.7 months. Only one patient discontinued the treatment because of toxicity [69]. In addition, a retrospective study revealed an ORR of 4.7% in 86 patients with metastatic UM who received pembrolizumab or nivolumab treatment, with a median OS of 14 months and 10 months, respectively [70]. Another retrospective review including 15 patients with metastatic UM showed no objective response to treatment (pembrolizumab or nivolumab), with a 3-month median PFS and 5-month median OS [71]. Recently, Koch et al. [56] reported an ORR of 8.9% among 45 patients with metastatic UM who received pembrolizumab or nivolumab treatment. In this retrospective study, 11 patients developed AEs and four developed severe AEs (grade 3 + 4) (Table 3) [56]. In fact, more valid data about PD-1 inhibitors were obtained from the IMCGp-100-202 trial, which contained the largest prospectively treated cohort (NCT03070392) [72]. About 80% of 126 patients in the control group received pembrolizumab treatment. The ORR was 5% and the DCR was 27%, with a median OS of 16 months and a PFS of 2.9 months [73]. Though a small number of patients in this group received ipilimumab or dacarbazine treatment, the clinical outcomes showed little difference from the trials stated above.

In regard to clinical practice, according to the results of Owen et al. [74], the effect of further therapy with PD-1 antibodies highly depended on the recurrence time: the earlier the recurrence, the poorer the response. As for those who have achieved a favorable response, however, it is still unknown how long PD-1 antibodies are supposed to be administered. Therapeutic strategies should be further investigated by longer observations. All in all, PD-1 or PD-L1 antibodies rarely induced continuous remission among patients with metastatic UM and only a few showed limited responses.

### 3.3. Combination of Anti-PD-1 and Anti-CTLA-4 Antibodies

Owing to the limited clinical response to antibody monotherapy, some studies have assessed the therapeutic effect of a combination of anti-PD-1 and anti-CTLA-4 antibodies. A phase II trial reported an ORR of 11.5% and SD of 51.9% among 52 patients with metastatic UM treated with nivolumab plus ipilimumab (NCT02626962) [75]. The median PFS and OS were 3.0 months and 12.7 months, respectively [75]. Another phase II trial achieved an ORR of 18% (one patient with CR and five with PR) in 33 evaluable patients who received nivolumab plus ipilimumab therapy (NCT01585194) [72]. It reported both longer median PFS (5.5 months) and median OS (19.1 months), yet with an incidence of severe AEs as high as 40% [72].

Several retrospective studies investigated treatment with anti-PD-1 in combination with anti-CTLA-4 antibodies and showed similar clinical outcomes. An earlier retrospective study suggested a median PFS of 2.8 months among 15 metastatic UM patients (12 evaluable) who received treatment with a PD-1 inhibitor and ipilimumab [70]. Similar outcomes were reported in two other studies, one of which included 64 patients with metastatic UM treated with nivolumab or pembrolizumab plus ipilimumab, whereas the other included 89 patients with metastatic UM treated with nivolumab plus ipilimumab [76,77]. The former reported a median PFS of 3 months and median OS of 16.1 months [76]. Meanwhile, 39.1% of patients developed severe AEs (grade 3, 37.5%; grade 4, 1.6%) [76]. The latter showed an ORR of 11.6%, median PFS of 2.7 months, median OS of 15 months, and incidence of severe AEs of 30% [77]. A retrospective population-based study enrolled 19 patients treated with combined ipilimumab and nivolumab [54]. The median PFS and median OS were 3.7 months and 18.9 months, respectively [54].

A retrospective case series of eight patients with metastatic UM assessed the efficacy of ipilimumab and nivolumab in combination with transarterial chemoembolization [78]. The median OS was 14 months and the median PFS was not reported [78].

To date, the largest retrospective multicenter study divided 178 patients with metastatic UM into two cohorts: A and B. Cohort A included 55 patients with only hepatic metastases and B contained 123 patients with both hepatic and other metastases [56]. Ninety-four patients (cohort A, *n* = 34; cohort B, *n* = 60) received combined treatment with anti-PD-1 and anti-CTLA-4. Moreover, 31.2% of patients developed severe AEs and there were no differences between cohorts A and B [56]. The entire cohort showed a median PFS of 2.8 months (cohort A, 2.4 months vs. cohort B, 2.9 months) and a median OS of 16 months (cohort A, 6.1 months vs. cohort B, 18.2 months) [56]. Although the two cohorts had similar median PFS rates, cohort B had a longer median OS than cohort A. Counterintuitively, patients developing both hepatic and other metastases responded better to dual ICB treatment and showed better survival than those with hepatic metastases only [56]. However, the reason for this therapeutic effect remains unclear and requires further investigation (Table 4).

As for the treatment-related adverse events (TRAEs) and treatment-related serious adverse events (TRSAEs), the two phase II trials showed similar toxic outcomes. Almost all patients experienced TRAEs, including diarrhea/colitis, fatigue, skin-related events, liver-related events, and hypothyroidism. TRSAEs occurred in about half of patients, including diarrhea, liver-related events, and fever [72,75]. Treatment-related deaths were rare, with reported cases including thyroiditis and Guillain−Barre syndrome [75]. However, the toxicity profile can be managed and shows little difference with that of CM, which makes it a potential therapeutic strategy [75].

The clinical outcomes of metastatic UM are inferior to that of metastatic CM. One reason is that both primary and metastatic UM carry an extremely low mutation burden compared to CM (a mean mutation rate of 0.5 vs. 49.2 mutations per megabase) [79,80]. This lower mutation rate may lead to poorer immune activity and less production of neoantigens [37]. Another reason is that the expression of PD-1/PD-L1 decreased more in UM metastases than in CM metastases [81]. Further, it was lymphocyte-activation gene 3 (LAG-3) that was detected as the dominant exhaustion marker, which could be another explanation of the limited effect of CTLA-4 and PD-1/PD-L1 inhibitors [82].

In summary, most patients who responded to ICI treatment only achieved PR, and the durability of the therapeutic benefit was limited. To date, neither the National Comprehensive Cancer Network (NCCN) nor the American Society of Clinical Oncology (ASCO) included any ICIs in their guidelines, although nivolumab and pembrolizumab were previously approved by the FDA as an adjuvant therapy in patients diagnosed with melanoma with lymph node involvement after complete resection of the tumor [83,84]. More high quality randomized controlled trials are warranted to further validate this therapeutic option, since there is a lack of data in prospective, large sample, evidence-based medicine. Nevertheless, this regimen, especially the combined anti-PD-1 and anti-CTLA-4 treatment, still has potential benefits, which may be considered in patients with otherwise limited options.

### 3.4. Potential Immune Checkpoints to Target

#### 3.4.1. T-Cell Immunoreceptor with Immunoglobulin and Immunoreceptor Tyrosine-Based Inhibitory Motif Domain Inhibitors

The immunoreceptor tyrosine-based inhibitory motif domain (TIGIT) is an inhibitory receptor on lymphocytes that can downregulate the functions of T and NK cells by interacting with CD155 expressed on APCs or tumor cells [85]. Chauvin et al. [86] found an upregulation of TIGIT and a co-expression of PD-1 in patients with melanoma. In addition, the expression of TIGIT could be further upregulated after PD-1 blockade. By blocking TIGIT and PD-1 receptors, they found an increase in degranulation and proliferation of CD8+ T-cells in the presence of cells expressing the TIGIT ligand [86]. Recently, Stalhammar et al. [87] discovered that primary tumors have a much higher mean number of TIGIT-positive cells/mm^2^ than normal choroid tissue, similar to the comparison between metastases and normal liver tissue. Metastatic primary UM has a higher number of TIGIT-positive cells/mm^2^ than non-metastatic UM and paired metastatic UM. Hence, it is reasonable to infer that TIGIT is a potential target for UM immunotherapy. Several monoclonal antibodies targeting TIGIT have been synthesized (iragolumab, AB-154, BMS-986,207, MK-7684) [88] and clinical trials on TIGIT inhibition in several cancer types (e.g., multiple myeloma and chronic myeloid leukemia) have been launched. Its effectiveness in advanced UM should be investigated in the future.

#### 3.4.2. IDO Inhibitors

Indoleamine 2, 3-dioxygenase is a rate-limiting metabolic enzyme able to convert tryptophan and affect proliferation, activation, and survival of lymphocytes [89,90]. Studies have indicated that IDO can inhibit T and NK cells and induce tumor angiogenesis [90]. In UM cells, IFN-γ-induced upregulation of IDO expression can lead to defense against T and NK cell immune responses, thus promoting immune escape [90]. Interestingly, combination therapy of IDO1 inhibitors with selected therapies always produces a more satisfactory clinical effect than IDO1 inhibitor monotherapy due to synergistic benefits [89]. A phase I/II trial demonstrated favorable antitumor activity and good tolerance to combined treatment with the IDO1 inhibitor epacadostat and pembrolizumab (ECHO-202/KEYNOTE-037), however, this trial did not include patients with UM [91]. Nevertheless, a phase III study showed that neither PFS nor OS improved in patients with unresectable or metastatic melanoma who received this treatment (NCT02752074) [92]. Stalhammar et al. [87] discovered that metastatic and nonmetastatic primary tumors have a higher mean number of IDO-positive cells/mm^2^ than normal tissue of the choroi, which is the same as metastases in comparison with normal tissue of the liver. The expression of IDO was associated with the expression of the checkpoint receptor TIGIT and both were moderately interrelated with the immune-related prognostic signature [87,93]. Overall, IDO might be a feasible immune checkpoint for the treatment of UM and several agents targeting IDO are currently being examined but do not include UM.

#### 3.4.3. LAG3

LAG3 is a receptor expressed on NK cells, T-cells, and plasmacytoid dendritic cells that has recently been recognized as an immune checkpoint [94,95]. Signals transduced by LAG-3 of T-cells can result in T-cell dysfunction and immune escape of tumors [95]. Woo et al. [96] found that LAG-3 and PD-1 were co-expressed on tumor-infiltrating lymphocytes (TILs) and had synergistic effects on the upregulation of the T-cell proportion and the maintenance of immune homeostasis. Blocking both receptors can slow tumor growth and strengthen antitumor immunity [96]. In patients with melanoma who progressed during previous anti-PD-1/PD-L1 therapy, combined treatment with the anti-LAG3 antibody (BMS-986016) and nivolumab showed clinical activity [97]. Regrettably, there is no clinical evidence of anti-LAG3 treatment in patients with UM. A single cell analysis of UM indicated that the expression level of exhaustion-associated immune checkpoint molecules on CD8+ T-cells was the highest for LAG3 but the lowest for PD1 [82]. Furthermore, the expression of LAG3 and its ligand Galectin-3 was positively correlated with high-risk clinical and histopathological features, including epithelioid cell type, loss of BAP1 staining, and monosomy of chromosome 3 [95]. These data indicate that LAG3 may be a potential checkpoint and it is reasonable to consider monoclonal antibodies against LAG3 as ICIs for the treatment of patients with UM.

## 4. Cancer Vaccines

### 4.1. Whole Cell-Based Vaccines

The rationale for whole cell-based vaccines is that tumor cells can serve as non-professional APCs and, surprisingly, can synthesize novel tumor antigen peptides from a non-traditional pathway, which is very different from the way professional APCs are synthesized [98,99]. Concerning UM cells, Verbik et al. [100] ascribed the suppression of CD8+ T-cell activation to the absence of HLA class II expression or co-stimulatory molecules. Hence, through genetic modification, UM cells can express recipient syngeneic MHC II (i.e., HLA II or HLA-DR) alleles and CD80 co-stimulatory molecules [101]. MHC II UM vaccines are prepared from these modified UM cells. Specifically, CD80 molecules can restrain the IFN-γ-mediated upregulated expression of PD-L1 and thus, solve the dilemma of T-cell suppression, while transduced MHC II molecules, owing to the lack of an MHC II-associated invariant chain (Ii), can bind atypical tumor peptides and facilitate antigen presentation via non-conventional intracellular trafficking patterns [99,102]. Significantly, although there is no need to match MHC I alleles, MHC II molecules must be paired with at least one allele in patients with UM [88].

The major objective of vaccination is to specifically activate CD4+ T-cells, as they are vital for both CD8+ T-cell-mediated protective immunity and immune memory [103,104]. First, they can act as classic “helper” T-cells to release multiple cytokines required by CD8+ T-cells [105,106]. Second, they promote dendritic cells (DCs) to express CD40 molecules (‘licensing’) and consequently, activate CD8+ T-cells [107,108]. Furthermore, CD4+ T-cells have a direct cytolytic effect on tumor cells, such as Fas-mediated cytolysis or tumor necrosis factor-related apoptosis-inducing ligand-induced apoptosis [109,110,111]. Vaccine-activated CD4+ T-cells can react with primary UM cells and cross-react with metastatic UM cells. Similarly, activated CD8+ T-cells also have a cytolytic function towards primary and metastatic UM cells [99,104].

In a recent study, Kittler et al. [112] expounded on Mel202/DR1/CD80 vaccines in detail. The cells of these vaccines primed and enhanced highly purified CD4+ T-cells; subsequently, activated cells proliferated, generated IFN-γ, and induced a polyclonal CD4+ T-cell repertoire, including T helper (Th) type 1, Th2, Th17, and T regulatory cells (Tregs) [112]. Among these, Treg cells seem to exert no distinct influence on the intensity of the anti-tumor vaccine response [112].

To date, clinical data have been restricted to exceedingly rare or anecdotal cases with favorable responses [98].

### 4.2. Dendritic Cell Vaccines

DCs, which have the most powerful antigen-presenting function, are the only professional APCs that can activate naïve antigen-specific T-cells. Therefore, it is appropriate to use DCs to induce immunologic antitumor responses and generate DC vaccines for treating stage IV melanoma [113]. In concrete terms, this method involves transfecting autologous monocyte-derived DCs with specific mRNA that encode target antigens in order to acquire optimized DC vaccines [98,114].

The tumor antigens gp100 and tyrosinase are expressed in most UM cells and thus constitute suitable targets for UM immunotherapy [35,36]. In a phase II study, Bol et al. [115] transfected DCs with mRNA encoding these two antigens to present HLA-A*02:01-restricted peptides and produced DC vaccines that could induce and stimulate the response of tumor-specific CD8+ or CD4+ T-cells. The median disease-free survival reached 34.5 months, and the 3-year OS rate of 79% among DC-vaccinated patients was also better than that reported in the literature (approximately 60% in high-risk UM). In addition, researchers have proposed that DC vaccination could induce de novo immune responses and was hypotoxic as an adjuvant treatment in high-risk UM patients [115]. To the best of our knowledge, there has been no clinical evidence indicating that DC vaccination immunotherapy is superior to other immune or non-immune treatments [14,116].

An ongoing randomized phase III study aimed to determine whether DC vaccines loaded with autologous tumor RNA can effectively prevent or delay UM progression in high-risk UM patients compared to standard care (NCT01983748). Another phase I trial aimed to examine the safety, tolerability, and OS prolongation of IKKb-matured, RNA-loaded DC vaccines in patients in with metastatic UM is ongoing (NCT04335890).

## 5. Cell Therapy

### 5.1. Adoptive Cell Transfer

The fundamental principle of adoptive cell transfer (ACT) is ex vivo activation and expansion of autologous immune cells and their subsequent reinfusion into patients [98]. The cells involved in this individualized immunotherapy are generally CD8+ T-cells or tumor-specific CD8+T-cells, but also include CD4+ Th cells. They can be derived from TILs isolated from tumor biopsies of patients with UM and can be engineered to target certain antigens [88]. Strobel et al. [117] have comprehensively reviewed this treatment option and thus, we briefly described it in this chapter. Additionally, we added some novel ongoing trials.

The regression of UM induced by the adoptive transfer of autologous TIL was first described by Chandran et al. [118,119]. Recently, several studies have started to introduce innovative ACT strategies. One was BPX-701, a T-cell product that transduced autologous T-cells with an HLA-A2-restricted PRAME-directed T-cell receptor (TCR) and an inducible caspase-9 safety switch (NCT02743611). PRAME, a preferentially expressed melanoma antigen, was expressed in approximately half of the primary and metastatic UM [120,121]. Another strategy involving autologous CD8 positive (+) SLC45A2-specific T-cells for SLC45A2 was expressed in 100% of UM cell lines but was very low or absent in normal tissues (NCT03068624). These T-cells, which are easily generated from donors, can kill the vast majority of HLA-matched melanoma cells [122]. The third was MAGE-C2/HLA-A2 TCR T-cells (NCT04729543). As a member of the cancer germline gene subfamilies, the Cancer Germline Antigen MAGE-C2 is only expressed in tumors, and this derived-antigenic peptide can induce targeted T-cell responses in some patients without detectable toxicity [123,124,125]. However, none of these studies reported the results.

### 5.2. Chimeric Antigen Receptor T-Cell Therapy

Chimeric antigen receptor (CAR) T-cell therapy is a burgeoning immunotherapy that has been used to treat various hematologic malignancies in recent years. This method involves infecting enriched T-cells with a retroviral vector (e.g., lentivirus) integrating necessary genetic information or inserting the desired gene modification via gene editing techniques, in order to achieve expression of CARs on T-cells [126]. These engineered T-cells are then expanded to the degree of clinical use [126]. As synthetic cell surface receptors, CARs generally consist of a target-binding extracellular domain, hinge region, transmembrane domain with an anchoring effect, and one or several intracellular signaling domains that activate T-cells [127,128]. Based on the combination of target-binding single chain variable fragments (scFv) with specifically intact surface antigens, CAR T-cells can recognize antigens without restrictions on the context of HLA molecules and are consequently considered widely suitable for HLA-diverse patient populations [128,129].

Forsberg et al. [130] confirmed that HER2 mRNA was the only molecule expressed at any appreciable level in the majority of UM among the most established CAR-T targets and UM could respond to HER2 CAR-T-cells in a target-specific manner. Uveal melanoma cells were killed by HER2 CAR-T-cells both in vitro and in human IL2 transgenic NOD/SCID IL2 receptor gamma knockout mice. These CAR-T-cells led to deep or complete regression of UM resistance to ACT therapy with autologous TILs, which would provide a novel treatment strategy and promote the clinical translation of CAR-T-cells. The therapeutic effects of HER2 CAR-T-cells have been proven in vivo and in vitro; its application in patients with UM and long-term effects require further studies.

An ongoing phase I study has employed C7R-GD2 CAR-T-cells to treat several GD2 positive solid cancers including GD2-positive UM. The study aimed to assess the tolerability, toxicity, and efficacy of C7R-GD2 CAR-T-cells (NCT03635632). Researchers have added the gene C7R on the basis of GD2 CAR-T-cells in order to supply these cells with constant and adequate cytokines and acquire longer survival times.

## 6. Immune-Mobilizing Monoclonal T-Cell Receptors against Cancer

Immune-mobilizing monoclonal T-cell receptors against cancer (ImmTACs) are a novel class of T-cells that redirect bispecific biologics and anti-tumor reagents [131]. These molecules, containing an affinity-enhanced soluble TCR connected to an optimized anti-CD3 scFv have a long binding half-life and are capable of redirecting T-cells to tumor cells with a low density of targets [131,132]. The high-affinity TCR can combine with the HLA/peptide complex present on the tumor cell surface, whereas anti-CD3 scFv ensures the recruitment of circulating T-cells and activation of TILs [133]. ImmTACs show clinical promise as well as theoretical advantages in mobilizing inflammatory cells and overcoming the tumor microenvironment in immune-suppressed (‘immunologically cold’) tumors [131].

As the first and only drug demonstrating clinical benefits in a phase III study, tebentafusp (also called IMCgp100) is an advanced ImmTAC molecule targeting HLA-A*02:01-restricted peptide gp100, which can kill targeted tumor cells by immune synapse [35,134]. It was developed from HLA-A*02:01, the most common HLA complex in humans. Different ethnic groups show different prevalences of HLA-A2. In Caucasians, Hispanics, and African Americans, the prevalence is 50%, 47%, and 35%, respectively [135]. Among these HLA-A2 positive groups, 96% of Caucasians, 59% of Hispanics, and 73% of African Americans present with HLA-A*02:01 [135]. Therefore, owing to HLA restriction, only part of the mUM population is eligible for tebentafusp treatment. Additionally, tebentafusp may generate significant toxicities in non-tumoral cells expressing the same peptide of HLA complexes [134].

In August 2021, tebentafusp was greenlit by both the European Medicines Agency (EMA) and the FDA for the treatment of HLA-A*02:01-positive adult patients with metastatic UM [136]. To date, three trials have reported the clinical results of tebentafusp in patients with UM: IMCGp-100-01 (NCT01211262), IMCGp-100-102 (NCT02570308), and IMCGp-100-202 (NCT03070392).

The IMCgp100-01 was the first in-class study of tebentafusp. It included 84 patients with advanced or metastatic melanoma (including a cohort of 16 patients with UM) (NCT01211262). Concerning UM, results indicated an ORR of 20% (three patients with PR) and a DCR of 67% in 15 evaluated patients, which jointly demonstrated the clinical activity of tebentafusp [137,138].

The IMCGp-100-102 was a phase I/II trial containing 146 pretreated patients with metastatic UM (NCT02570308). As for the phase I cohort performed on 19 heavily pretreated patients, a DCR of 47.4% and ORR of 15.8% (three PR) were reported, together with a median OS of 29.6 months [139,140]. The phase II cohort, however, showed a DCR of 22.8% and an ORR of 4.7% among 127 pretreated patients [140]. The median PFS and OS achieved were 2.8 months and 16.8 months, respectively, which were profoundly better than the documented results in previous trials [134].

The IMCGp-100-202 was a phase III trial, which contained a control group of investigator’s choice of therapy (ICOT) and an experimental group of tebentafusp in a 1:2 ratio (NCT03070392) [73]. Due to the absence of a standard-of-care systemic therapy, 126 untreated patients with metastatic UM in the ICOT group were administered pembrolizumab (82%), ipilimumab (13%), or dacarbazine (6%) [141]. The median OS was 21.7 months (95% CI: 18.6–28.6) in EG compared to 16.0 months (95% CI: 9.7–18.4) in CG [73,141]. The median PFS was 3.3 months (95% CI 3.0–5.0) in EG versus 2.9 months (95% CI 2.8–3.0) in CG [73] (Table 5). The primary endpoint of this trial has been achieved; in this context, researchers demonstrated that severe AEs, which developed in 44% and 17% of patients in the two groups, respectively, were hardly affected with continued treatment. Meanwhile, the most frequent AEs were divided into two categories: cytokine-release syndrome (88% of grade 1–2 AEs; 1% of grade 3 AEs) and rash (65% of grade 1–2 AEs; 18% of grade ≥ 3 AEs) [140,141].

More recently, a phase I study identified 68 mg as the recommended phase II dose of tebentafusp by a three-week step-up dosing regimen, which was 36% higher than the maximum tolerated dose of the first in-human trial [138]. Forty-two HLA-A*02 or HLA-A*02:01-positive patients with metastatic UM showed a 67% 1-year OS rate and 25.5 months of median OS, suggesting the clinical antitumor activity of this novel regimen [138]. Currently, an expanded access program is ongoing (NCT04960891) to provide access to tebentafusp for patients with metastatic UM. Another phase II non-randomized study was designed to identify the safety and efficacy of tebentafusp in molecular relapsed disease melanoma (including CM and UM) (NCT05315258).

## 7. Oncolytic Virus

Oncolytic viruses are a type of replicating viruses characterized by an engineered or intrinsic tumor-specific lytic function [143,144]. Previous studies have illustrated that viral administration significantly strengthens both innate and adaptive immune responses and overcomes the immunosuppressive mechanisms of tumors [145]. Oncolytic viruses are currently being studied in cancer and have shown good tolerability with no severe toxicity [146]. It also has the particular advantage of targeting and killing tumor cells with high selectivity, thus sparing normal cells [147]. Herein, we describe oncolytic viruses adapted from herpes simplex virus type 1 (HSV-1), enteric cytopathic human orphan virus type 7, vesicular stomatitis virus (VSV), and oncolytic adenovirus (Ad).

### 7.1. HSV-1

The modified HSV-1 armed with GM-CSF (HSV-GM-CSF) has the same structure as T-VEC (Imlygic^®^, Amgen). As the first oncolytic immunotherapy showing therapeutic benefits for melanoma, it was approved by the FDA as a local therapy for unresectable melanoma [148,149,150,151]. Liu et al. [148] indicated that GM-CSF treatment boosted the antitumor effect of HSV-1 in vitro and in vivo and induced the expression of GM-CSF and the infiltration of macrophages. As the research revealed, the tumor volume was reduced and the median survival time was prolonged in mouse models of the HSV-GM-CSF group [148].

Oncolytic HSV-EGFP has the potential to be another effective, immune-active, and safe option for UM treatment [152]. In the systemic UM xenograft model, the tumor size was reduced in both local tumors injected with oncolytic HSV-EGFP and remote subcutaneous tumors without injection. More importantly, the immune microenvironment was substantially changed, mainly manifested by the increased expression of the macrophage-related factor IFN-γ and shifted the macrophage polarization from the M2 phenotype to the M1 phenotype [153].

### 7.2. VSV

A study conducted by Wollmann et al. [154] found that most human melanoma types were susceptible to VSV-mediated oncolysis and that VSV tended to infect and kill melanoma cells rather than normal melanocytes. VSV encoding the IFNβ transgene (VSV-IFNβ) is capable of preventing tumor growth through multiple mechanisms, such as triggering direct cell killing, stimulating innate immune response, recruiting CD8 T-cells, and depleting T-regulatory cells [155].

Currently, a phase I trial based on previous findings is ongoing, with the aim to evaluate the safety and efficacy of VSV-expressing human IFNβ and tyrosinase-related protein 1 (VSV-IFNβ-TYRP1) in patients with metastatic UM and CM (NCT03865212).

### 7.3. Oncolytic Adenovirus

Several major capsid proteins of Ad and its non-coding RNAs play critical roles in synergistically activating the innate immune system, resulting in inflammation and elimination of its vector [156]. In addition, Ad vector administration can also induce maturation of DCs and activation of T-cells, consequently eliciting humoral and cellular immune responses in hosts [157].

Evidence has shown that the oncolytic adenovirus ICOVIR-5 was able to sustain valid anti-tumoral activity and replicated preferentially in tumor cells with pRB pathway dysregulation [158]. A phase I trial in patients with CM and UM suggested that intravenous administration of ICOVIR-5 could not induce regression of the tumor [159]. Despite no tumor response, seven of the 11 patients achieved a stable disease state [159].

Oncolytic adenovirus H101 is a clinical treatment with favorable tolerance and efficacy and has been approved by the Chinese State Food and Drug Administration for the treatment of several malignancies [160]. The first trial of UM cell lines in vitro demonstrated that combined H101 with the alkylating agent dacarbazine led to a cell cycle blockade and played a synergistic antitumor role in killing UM cells [161].

Another trial on the combination of H101 with small interfering RNA (siGNAQ) demonstrated a potential clinical utility among UM cells with GNAQ mutations [162]. The apoptotic rate increased significantly in the combined treatment group, confirming that the apoptosis-inducing activity of H101 could be enhanced by combining it with siGNAQ. Cell cycle distribution was also altered to prominent and extensive G0/G1 phase arrest with combined treatment.

Concerning other oncolytic viruses, an open-label phase Ib clinical study combining ipilimumab with intravenous oncolytic virus CAVATAK^®^ (Coxsackievirus A21, CVA21) is ongoing in patients with liver metastases of UM (NCT03408587).

## 8. Combinations of Immunotherapy with Small Molecular Inhibitors

Because of the limited clinical outcomes of various monotherapies such as ipilimumab, pembrolizumab, and nivolumab, researchers have explored new immunotherapy strategies of combination treatments with small molecular inhibitors and these attempts have made certain progress.

### 8.1. Histone Deacetylase Inhibitors and Their Combined Treatments

The balance between histone acetylation and deacetylation is crucial for regulating gene expression [163]. Induced by histone acetyltransferase and HDAC, acetylation and deacetylation of histones are related to gene transcription and silencing, respectively [164]. Specifically, HDACs are capable of regulating the expression of tumor suppressor genes and activity of transcriptional factors associated with the initiation and development of tumors by transforming the chromatin structure [165]. The inhibition of HDAC causes apoptosis, growth arrest, and inhibition of angiogenesis [166]. In UM, HDAC inhibitors can lead to the differentiated and quiescent state of UM cells, cell-cycle exit, and clinical dormancy of tumors and they increase HLAI expression [167,168]. Several HDAC inhibitors, such as trichostatin A and tenovin-6, show promising therapeutic effects against UM in vitro and/or in vivo [164]. Previous reviews have summarized these in detail; here, we mainly focus on recent studies.

A study showed that combined treatment with the ERBB1/2/4 inhibitor neratinib and the HDAC inhibitor entinostat improved MHCA levels and reduced the expression of immunological biomarkers, including PD-L1, IDO-1, and ODC in six hours [169]. This combination was able to downregulate the expression of K-RAS V12, Gαq, and Gα11, and also initiate the death of UM cells by mitochondrial dysfunction and toxic autophagy [169].

Another phase II study reported the clinical effects in 29 patients with metastatic UM treated with the HDAC inhibitor, entinostat, in combination with the PD1 inhibitor, pembrolizumab (NCT02697630) [170,171]. The ORR was 14% (four patients with PR), with a median PFS of 2.1 months and a median OS of 13.4 months [171]. With regard to safety, 19 patients (66%) developed severe AEs, although no treatment-related deaths were reported [171]. A recent study also proved that entinostat enhanced the anti-tumoral functions of T-cells and combining entinostat with a PD1 inhibitor slowed down tumor growth and extended survival [172].

Inhibitors of HDAC are promising treatments for patients with UM and are worth evaluating in further studies.

### 8.2. Poly (ADP-Ribose) Inhibitors and Their Combined Treatments

BRCA1-associated protein 1 (BAP1) can be used to facilitate DNA repair and survival after damage and its mutation, which is associated with a high risk of metastases, can result in defects in this function [173,174]. In patients with UM, especially those carrying BAP1 mutations (over 80% as reported), the survival of UM cells and the repair of DNA consequently depends on parallel DNA repair pathways such as nucleotide excision repair and base-excision repair, in which poly (ADP-ribose) polymerase (PARP) plays an essential role [175,176]. Studies have demonstrated that a DNA repair deficiency caused by PARP inhibitors may sensitize tumors to the immune response [177]. Meanwhile, damaged DNA released in the cytosol can induce an immune response via activation of specific pathways (e.g., interferon gene (STING) pathway) and accumulation of abnormal mutations [177,178]. High expression of PARP-1 is associated with poorer OS and has been found to be an adverse prognostic factor in UM [179].

A preclinical trial showed that monotherapy with the PARP inhibitor olaparib was not efficient; it greatly enhanced the efficacy of the alkylating agent dacarbazine [173]. This possible synergy could be of clinical significance in treating UM [173]. A recent study also demonstrated that olaparib significantly regulates the expression of 20 long non-coding RNAs in UM and probably further influences related transcription factors and subsequent target genes [180].

Studies have also shown that complicated DNA damage caused by PARP inhibitors could influence tumor immunogenicity, including upregulation of PD-L1 expression, and consequently boost the effect of ICIs [181,182]. Based on this, combined PARP inhibitors with ICIs have been investigated in several trials of solid tumors (e.g., ovarian cancer, prostate cancer, and breast cancer) with favorable results as well as toxicity profile [183]. Although UM is not currently used in such studies, it can be a feasible candidate for this combination.

## 9. Conclusions and Outlook

To date, many treatments for patients with UM have shown disappointing clinical outcomes and patients still suffer from unfavorable long-term prognoses. This review mainly focused on newly published research on immunotherapy for UM. ICIs showed limited effects; however, several new targeted checkpoints showed therapeutic feasibility. Cancer vaccines and adoptive cell transfer seem promising but require further study. Tebentafusp has been intensively studied in recent years, and has demonstrated both feasible and optimistic results. Furthermore, the combination of oncolytic viruses and certain agents improved the therapeutic effect to varying degrees.

Although new effective therapeutic strategies have been developed recently, more details about UM tumor genesis mechanisms still need to be explored. First, it is necessary to have more comprehensive knowledge of oncogenic events of UM and their sequences and the biological consequences to prevent or treat UM metastases. In addition, similar information on genetic variations in both primary and metastatic UM may help in designing treatments for patients with metastases [1,184]. Meanwhile, additional mutations in metastatic UM probably indicate the emergence of therapeutic targets, which implies that treatments that lead to DNA damage and result in increased mutational burden may be feasible [1]. Further investigation of the microenvironment of UM, clarity of its relevant immune cells and immune escape mechanisms, determination of effective therapeutic strategies, and exploration of potential immune checkpoints to overcome the current situation of poor immunotherapy effect are needed. It is essential to treat metastatic UM and we should encourage more patients to participate in future trials. We hope that ongoing trials can provide favorable results and ultimately achieve our goals of protecting vision, controlling UM metastasis, and, most importantly, saving lives.

## Figures and Tables

**Table 1:** Clinical studies with CTLA-4 inhibitors in metastatic UM patients.

Study Type	Therapy	Patients Enrolled	ORR	DCR	mPFS	mOS	Reference
Multicenter, retrospective	Ipilimumab	20	NR	NR	NR	5 months	[53]
Retrospective, Population-based	Ipilimumab	24	0	25%	3 months	9.9 months	[54]
Phase Ib/II	0.3 mg/kg ipilimumab + RFA	3	0	NR	3 months	NR	[55]
	3 mg/kg ipilimumab + RFA	19	0	11%	3 months	9.7 months	
	10 mg/kg ipilimumab + RFA	19	0	5%	3 months	14.2 months	
	Total	41 (37 evaluable)	0	7%	3 months	12.4 months	
Multicenter, retrospective	Ipilimumab	11	0	18.2%	NR	NR	[56]
Phase II	Tremelimumab	11	0	NR	2.9 months	12.8 months	[57]

ORR, overall response rate; DCR, disease control rate; mPFS, median progression-free survival; mOS, median overall survival; NR, not reported; RFA, radiofrequency ablation.

**Table 2:** Ongoing clinical trials with immunotherapies in UM and metastatic UM.

NCT Number	Study Title	Status	Phase	Conditions	Population
NCT05315258	Tebentafusp in Molecular Relapsed Disease (MRD) Melanoma	Not yet recruiting	Phase 2	Skin Melanoma Uveal Melanoma	Enrollment: 50
NCT05308901	Lenvatinib Plus Pembrolizumab in Patients with Immune Checkpoint Inhibitor Naïve Metastatic Uveal Melanoma	Not yet recruiting	Phase 2	Uveal Melanoma	Enrollment: 30
NCT05282901	Efficacy and Safety of Pembrolizumab in Combination with Lenvatinib in Metastatic Uveal Melanoma Patients (PLUME)	Not yet recruiting	Phase 2	Metastatic Uveal Melanoma	Enrollment: 54
NCT05077280	A Study of Concurrent Stereotactic Body Radiotherapy with Ipi and Nivo in Metastatic Uveal Melanoma	Recruiting	Phase 2	Uveal Melanoma	Enrollment: 40
NCT04960891	A Cohort IND Expanded Access Program for Supporting Patient Access to Tebentafusp	Available		Uveal Melanoma	
NCT04935229	Intrahepatic Delivery of SD-101 by Pressure-Enabled Regional Immuno-oncology (PERIO), with Checkpoint Blockade in Adults with Metastatic Uveal Melanoma	Recruiting	Phase 1	Metastatic Uveal Melanoma in the Liver	Enrollment: 80
NCT04812470	Hepatic Arterial Infusion of Autologous Tumor Infiltrating Lymphocytes in Patients with Melanoma and Liver Metastases	Not yet recruiting	Phase 1	Metastatic Uveal Melanoma Metastatic Cutaneous Melanoma	Enrollment: 6
NCT04729543	MAGE-C2 TCR T Cell Trial to Treat Melanoma and Head and Neck Cancer	Recruiting	Phase 1 Phase 2	Melanoma Uveal Melanoma Head and Neck Cancer	Enrollment: 20
NCT04552223	Nivolumab Plus Relatlimab in Patients with Metastatic Uveal Melanoma	Recruiting	Phase 2	Metastatic Uveal Melanoma	Enrollment: 27
NCT04463368	Isolated Hepatic Perfusion in Combination with Ipilimumab and Nivolumab in Patients with Uveal Melanoma Metastases	Recruiting	Phase 1	Uveal Melanoma Liver Metastases	Enrollment: 18
NCT04335890	IKKb-matured, RNA-loaded Dendritic Cells for Metastasised Uveal Melanoma	Active, not recruiting	Phase 1	Metastatic Uveal Melanoma	Enrollment: 12
NCT04283890	PHP and Immunotherapy in Metastasized UM	Recruiting	Phase 1 Phase 2	Metastatic Uveal Melanoma	Enrollment: 83
NCT03922880	Study of Immunotherapy Plus ADI-PEG 20 for the Treatment of Advanced Uveal Melanoma	Active, not recruiting	Phase 1	Uveal Melanoma	Enrollment: 9
NCT03865212	Modified Virus VSV-IFNbetaTYRP1 in Treating Patients with Stage III-IV Melanoma	Suspended	Phase 1	Clinical Stage III Cutaneous Melanoma AJCC v8 Clinical Stage IV Cutaneous Melanoma AJCC v8 Metastatic Choroid Melanoma and 10 more	Enrollment: 72
NCT03635632	C7R-GD2.CART Cells for Patients With Relapsed or Refractory Neuroblastoma and Other GD2 Positive Cancers (GAIL-N)	Recruiting	Phase 1	Relapsed Neuroblastoma Refractory Neuroblastoma Uveal Melanoma and 4 more	Enrollment: 94
NCT01585194	Nivolumab and Ipilimumab in Treating Patients with Metastatic Uveal Melanoma	Active, not recruiting	Phase 2	Metastatic Uveal Melanoma Stage IV Uveal Melanoma AJCC v7	Enrollment: 67
NCT00471471	Vaccine Therapy in Treating Patients with Recurrent Stage III or Stage IV Melanoma That Cannot Be Removed by Surgery	Completed	Phase 1	Intraocular Melanoma Malignant Conjunctival Neoplasm Skin Melanoma	Enrollment: 22
NCT00398073	Vaccine Therapy in Treating Patients With Stage IIB, Stage IIC, Stage III, or Stage IV Melanoma	Completed	Phase 1	Intraocular Melanoma Skin Melanoma	Enrollment: 35
NCT00334776	Vaccine Therapy in Treating Patients with Metastatic Melanoma	Completed	Phase 2	Intraocular Melanoma Skin Melanoma	Enrollment: 6
NCT00313508	Dendritic Cell Vaccination During Lymphoid Reconstruction	Completed	Phase 1	Intraocular Melanoma Skin Melanoma	Enrollment: 18
NCT03611868	A Study of APG-115 in Combination with Pembrolizumab in Patients with Metastatic Melanomas or Advanced Solid Tumors	Recruiting	Phase 1 Phase 2	Unresectable or Metastatic Melanoma or Advanced Solid Tumors Melanoma Uveal Melanoma and 11 more	Enrollment: 224
NCT03472586	Ipilimumab and Nivolumab with Immunoembolization in Treating Participants with Metastatic Uveal Melanoma in the Liver	Active, not recruiting	Phase 2	Metastatic Malignant Neoplasm in the Liver Metastatic Uveal Melanoma Stage IV Uveal Melanoma AJCC v7	Enrollment: 35
NCT03467516	Adoptive Transfer of Tumor Infiltrating Lymphocytes for Metastatic Uveal Melanoma	Recruiting	Phase 2	Uveal Neoplasms Uveal Melanoma	Enrollment: 47
NCT03408587	CAVATAK^®^ and Ipilimumab in Uveal Melanoma Metastatic to the Liver (VLA-024 CLEVER)	Completed	Phase 1	Uveal Melanoma Liver Metastases	Enrollment: 11
NCT03070392	Safety and Efficacy of IMCgp100 Versus Investigator Choice in Advanced Uveal Melanoma	Active, not recruiting	Phase 2	Uveal Melanoma	Enrollment: 378
NCT03068624	Autologous CD8+ SLC45A2-Specific T Lymphocytes with Cyclophosphamide, Aldesleukin, and Ipilimumab in Treating Patients with Metastatic Uveal Melanoma	Recruiting	Phase 1	Metastatic Malignant Neoplasm in the Liver Metastatic Uveal Melanoma	Enrollment: 30
NCT03025256	Intravenous and Intrathecal Nivolumab in Treating Patients with Leptomeningeal Disease	Recruiting	Phase 1	Melanocytoma Metastatic Melanoma Metastatic Uveal Melanoma and 9 more	Enrollment: 50
NCT02913417	Yttrium90, Ipilimumab, & Nivolumab for Uveal Melanoma with Liver Metastases	Active, not recruiting	Phase 1 Phase 2	Uveal Melanoma Hepatic Metastases	Enrollment: 26
NCT02743611	Safety & Activity of Controllable PRAME-TCR Therapy in Previously Treated AML/MDS or Metastatic Uveal Melanoma	Unknown status	Phase 1 Phase 2	Acute Myeloid Leukemia Myelodysplastic Syndrome Uveal Melanoma	Enrollment: 28
NCT02697630	Efficacy Study of Pembrolizumab with Entinostat to Treat Metastatic Melanoma of the Eye	Active, not recruiting	Phase 2	Metastatic Uveal Melanoma	Enrollment: 29
NCT02626962	Trial of Nivolumab in Combination with Ipilimumab in Subjects with Previously Untreated Metastatic Uveal Melanoma	Completed	Phase 2	Uveal Melanoma	Enrollment: 52
NCT02570308	A Study of the Intra-Patient Escalation Dosing Regimen with IMCgp100 in Patients with Advanced Uveal Melanoma	Active, not recruiting	Phase 1 Phase 2	Uveal Melanoma	Enrollment: 146
NCT02519322	Neoadjuvant and Adjuvant Checkpoint Blockade	Active, not recruiting	Phase 2	Stage IIIB Uveal Melanoma AJCC v7 Stage IIIC Uveal Melanoma AJCC v7 Stage IV Uveal Melanoma AJCC v7 and 8 more	Enrollment: 53
NCT02359851	Pembrolizumab in Treating Patients with Advanced Uveal Melanoma	Terminated	Phase 2	Stage IIIA Uveal Melanoma Stage IIIB Uveal Melanoma Stage IIIC Uveal Melanoma Stage IV Uveal Melanoma	Enrollment: 5
NCT02158520	Nab-Paclitaxel and Bevacizumab or Ipilimumab as First-Line Therapy in Treating Patients with Stage IV Melanoma That Cannot Be Removed by Surgery	Completed	Phase 2	Stage IV Cutaneous Melanoma AJCC v6 and v7 Stage IV Uveal Melanoma AJCC v7 and 3 more	Enrollment: 24

**Table 3:** Clinical studies with PD-1/PD-L1 inhibitors in metastatic UM patients.

Study Type	Therapy	Patients Enrolled	ORR	DCR	mPFS	mOS	Reference
Single institution retrospective	Nivolumab	14	7.1%	42.9% (1PR,5SD)	10 weeks	60 weeks	[62]
Multicenter, retrospective	Nivolumab	17	18%	50% (1CR,2PR,5SD)	5.8 months	10.5 months	[63]
Single arm, phase II	Pembrolizumab	5	20%	60% (1CR,2SD)	11 months	NR	[66] NCT02359851
Prospective observational cohort single arm	Pembrolizumab	17	11.7%	47% (2PR,6SD)	3.8 months	NR	[67]
Retrospective population-based	Pembrolizumab	43	7%	35% (3PR,12SD)	4.8 months	10.3 months	[54]
Single center, prospective	Pembrolizumab	9	0	56% (5SD)	18 weeks	46 weeks	[68]
Retrospective	Pembrolizumab	38	2.6%	13.2% (1PR,4SD)	NR	NR	[69]
	Nivolumab	16	6.3%	12.5% (1PR,1SD)	NR	NR	
	Atezolizumab	2	0	0	NR	NR	
	Total	56	3.6%	12.5% (2PR,5SD)	2.6 months	7.7 months	
Retrospective	Pembrolizumab	54	5.7%	22.6% (3PR,9SD)	3.1 months	14 months	[70]
	Nivolumab	32	3.1%	18.7% (1PR,5SD)	2.8 months	10 months	
	Total	86	4.7%	20.9% (4PR,14SD)	NR	NR	
Retrospective	Pembrolizumab or nivolumab	15	0	26.7% (4SD)	3 months	5 months	[71]
Multicenter, retrospective	Pembrolizumab or nivolumab	45	8.9%	28.9% (4PR,9SD)	NR	NR	[56]

ORR, overall response rate; DCR, disease control rate; mPFS, median progression-free survival; mOS, median overall survival; NR, not reported; CR, complete response; PR, partial response; SD, stable disease.

**Table 4:** Clinical studies with combined PD-1 and CTLA-4 inhibitors in metastatic UM patients.

Study Type	Therapy	Patients Enrolled	ORR	DCR	mPFS	mOS	Reference
Single arm, phase II	Nivolumab and ipilimumab	52	11.5%	63.5% (1CR,5PR,27SD)	3 months	12.7 months	[75] NCT02626962
Single arm, phase II	Nivolumab and ipilimumab	35 (33 evaluable)	18%	51.5% (1CR,5PR,11SD)	5.5 months	19.1 months	[72]
Retrospective	PD-1 inhibitor and ipilimumab	15 (12 evaluable)	16.7%	33.3% (2PR,2SD)	2.8 months	NR	[70]
Multicenter, retrospective	Nivolumab/pembrolizumab and ipilimumab	64	15.6%	37.5% (2CR,8PR,14SD)	3 months	16.1 months	[76]
Multicenter, retrospective	Ipilimumab and nivolumab	89	11.6%	36% (1CR,9PR,21SD)	2.7 months	15 months	[77]
Retrospective population-based	Ipilimumab and nivolumab	19	21.1%	31.6% (4PR,2SD)	3.7 months	18.9 months	[54]
Single center, retrospective	Ipilimumab and nivolumab in combination with TACE	8	25%	75% (2PR,4SD)	NR	14 months	[78]
Multicenter, retrospective	PD-1 inhibitor and CTLA-4 inhibitor (dual ICI)	Cohort A (liver metastases only) 34	8.7%	35.3% (3PR,9SD)	NR	NR	[56]
		Cohort B (several metastatic sites) 60	16.7%	43.3% (10PR,16SD)	NR	NR	
		Total 94	13.8%	40.4% (13PR,25SD)	NR	NR	

ORR, overall response rate; DCR, disease control rate; mPFS, median progression-free survival; mOS, median overall survival; NR, not reported; CR, complete response; PR, partial response; SD, stable disease; TACE, transarterial chemoembolization; ICI, immune checkpoint inhibitor.

**Table 5:** Clinical trials with tebentafusp in metastatic UM patients.

Clinical Trial	Design	Therapy	Patients Enrolled	DCR	ORR	OS	PFS
IMCgp-100-01 [142]	Phase I	Tebentafusp	Heavily pretreated	67%	20%	1Y-OS: 65%	NR
NCT01211262			16			mOS: 33.4 months	
IMCGp-100-102 [142]	Phase I	Tebentafusp	Heavily pretreated	47.4%	15.8%	1Y-OS: 74%	7.4 months
NCT02570308			19			mOS: 29.6 months	
	Phase II	Tebentafusp	Pretreated	22.8%	4.7%	1Y-OS: 62%	2.8 months
			127			mOS: 16.8 months	
IMCGp-100-202 [72]	Phase III	CG: ICOT	Untreated	27%	5%	1Y-OS: 59%	2.9 months
NCT02570308			126			mOS: 16.0 months	
		EG: Tebentafusp	Untreated	46%	9%	1Y-OS: 73%	3.3 months
			252			mOS: 21.7 months	

DCR, disease control rate; ORR, overall response rate; OS, overall survival; PFS, progression-free survival; NR, not reported; mOS, median overall survival; ICOT, investigator’s choice of therapy; CG, control group; EG, experimental group; Y, year.
